# Supplementary material for: A multi-attribute method for ranking influential nodes in complex networks
Source: PLoS One. 2022 Nov 28;17(11):e0278129. doi: 10.1371/journal.pone.0278129 (PMC9704601; doi:10.1371/journal.pone.0278129)
Supplement: Attachment — Submitted filename: Response to Reviewers.docx [file pone.0278129.s001.docx]

**Manuscript Ref. No.: PONE-D-22-15682**

Dear Editor,

**Reply to reviewers on** PONE-D-22-15682

We would like first to thank the editor and the reviewers for helpful, constructive comments and observations that significantly contributed to improving the manuscript. Below we have concisely addressed each of the comments and suggestions and those changes implemented in the manuscript.

Also, we would like to note that the changes made in the text have been highlighted in blue in the new version of the manuscript to allow the reviewers to identify the newly added text easily.

Below you can find detailed explanations regarding each particular comment and the applied revision in the revised version of the manuscript.

Yours Sincerely,

Amir Sheikhahmadi, PhD

Assistant Professor, Islamic Azad University,

Sanandaj branch, Sanandaj, Iran

Email: sheikhahmadi@eng.ui.ac.ir

Reviewers' comments:

Reviewer #1:

The contribution of this paper is good and I am happy to endorse its acceptance at some point. However, there are several major and minor comments to address.

**Authors reply:** We thank the reviewer for taking the time to read the manuscript and providing positive comments. We have tried our best to reply to your comments below.

1. First off, please clearly state the gap targeted in this paper at the end of introduction and list down the hypotheses

**Authors Reply:** Thanks for your valuable comments. Items have been added to the introduction section**.**

2. In terms of research method and design, there is not much in the paper.

**Authors Reply:** proper changes were done in **the introduction and related works sections.**

3. The comparative algorithms in the experiments are not properly acknowledged and cited.

**Authors reply:** Thanks for your consideration. All of the methods were added and referenced in the literature review section.

4. I also suggest adding some figures to better articular the content as the paper looks very dry at the moment.

**Authors reply: I agree entirely. For better understanding, several figures were added in different sections.**

5. Analysis of the results is missing in the paper. There is a big gap between the results and conclusion. There should be the result analysis between these two sections. After comparing the numerical methods, you have to be able to analyse the results and relate them to their structures. It would be interesting to have your thoughts on why the method works that way? Such analyses would be the core of your work where you prove your understanding of the reason behind the results. You can also link the findings to the hypotheses of the paper. Long story short, this paper requires a very deep analysis from different perspectives.

There is no statistical test to judge about the significance of the numerical method’s results. Without such a statistical test, the conclusion cannot be supported.

**Authors reply:**

Thanks for your careful reading. Two new sections have been added to the evaluations for comparison and analysis. A portion of the tests is dedicated to testing the validity of the top-ranked nodes on the ranked list because those nodes are more important than those at the bottom. To accomplish this, the similarity c of the initial element of the list R ranked by each method with the ranking list of the actual publication σ is calculated.

6. There is no discussion on the cost effectiveness of the proposed method. What is the computational complexity? What is the runtime? Please include such discussions. You can also use the big oh notation to show the computation complexity.

**Authors reply:** In the evaluation section, a comparison of the execution time of the proposed method compared to other added methods.

7. Some mathematical notations and Lemma presentations are not rigorous enough to correctly understand the contents of the paper. The Authorss are requested to recheck all the definition of variables and further clarify these equations.

**Authors reply:** Thank you, all the items were reviewed and corrected

Reviewer #2:

In this paper the Authorss propose a method to determine and rank node diffusion powers using various attributes. In my opinion, the paper is interesting but it is not well structured and there are some weaknesses that must be solved.

**Authors reply:** Thanks for your valuable comments. We have tried our best to reply to your comments below.

1. The abstract must be rewritten because of it do not is a summary of the paper. It is a copy of a part of the introduction.

**Authors reply:** Thanks for your consideration. The abstract was modified.

2. Regarding to section 1 (Introduction), It does not explain the motivation that leads the Authorss to carry out the paper. Why is the article written? What does it mean that they don't have other articles already written? In the introduction, they insist that the accuracy is increased, based on what? I think you can't say that without saying why. Also, when they comment on the three elements of innovation of the study, suddenly ELECTRE appears without reference or anything, what is that?

**Authors reply:** Thanks, the introduction section was modified and the requested items added.

3. I don't understand this section, why are there two subsections? Why are seven measures chosen? Aren't there more? Are these seven metrics going to be used to make a comparison? In 2.2 subsection the paper says that there are two methods to analyze the diffusion power of nodes in social networks. All the time are talking about diffusion power in complex networks and suddenly they are limited to social networks, I don't understand. In addition, they say that one method is to calculate the node diffusion power in a real manner, I am not clear that this is a method, it is a kind of brute force to analyze the network and it is not used.
   There are many different diffusion evaluation methods: the cascade method, the linear threshold method, the SIR method, etc. Why do they use the SIR model? Why is it the most famous?

**Authors reply:** Regarding the use of the SIR model, it should be noted that this model is a generalized IC model, where the probability of infected nodes improving at the end of each stage is 1. However, in the linear threshold model, a threshold is needed to activate each node, and because this threshold is generally unavailable, the SIR model has been used in many articles and utilized in our study.

4. In the propose method section a flowchart appears, which is very important, but there are elements that need to be changed or better explained. Again the ELECTRE method appears without explanation. What does it consist of? Is it a James Bond method :)) ?
   Many methods use network structures to calculate the node powers. Why the Authorss use these ten methods? Also, some of them have not been explained in Literature review, there is a contradiction.
   Another important thing is that they suddenly remove Betweenness and Closeness basically due to their complexity on large networks. But it is obvious that there are methods for calculating Betweenness with lower computational cost, even without calculating the shortest path. It seems that they are eliminated by superficial criteria. Also, if they are not going to be chosen, it would not have been better not to put them in section 3.2 and the problem is over.
   The Authorss base the selection on the correlation, using the Spearman coefficient, between the centrality measures and the result of the SIR model. I'm sure if other flow based measures are used the correlation would be higher, which leads me to think that the result of the method they present depends on the methods used.

**Authors reply: Your consideration and excellent vision are respectable. The proposed method was altered based on your valuable comments. Closeness and Betweenness attributes were removed.**

5. This part seems to be the best part of the paper and I don't think many changes would be needed. In fact, until I have reached this part I have understood almost nothing. In short, I believe that the paper is novel and has the potential to be published, but it suffers from a lack of clarity, especially in the first sections.

Authors reply: Thanks for your valuable comments. Initial sections were changed and two new comparisons were added to the evaluation section as well.

Reviewer #3:

This paper has a potential to be accepted, but some important points have to be clarified or fixed before we can proceed and a positive action can be taken.

**Authors reply:** Thanks for valuable comments. We have tried our best to reply your comments in below.

1. The manuscript needs extensive English editing because there are several typos and grammatical errors.

**Authors reply**: Thanks.All sections have been revised.

2. It is really unclear to me why the Authorss applied SIR diffusion model. What is the specific property of this model?
**Authors reply: This is an excellent suggestion to be used in future works. In this study, the implementation of the SIR model with many iterations to calculate the propagation power of nodes can indicate the reality of propagation in networks~~,~~ . this model has been used in many articles.**

3. The literature review section is very poor. There are several descent research efforts that can be considered in this section.

**Authors reply**: The literature reviews completely modified and new references, added to this section.

4.The legends in Fig. 5 are missing.

**Authors reply**: Thanks, It is modified .

5.I'm not convinced that high correlation of an attribute with the diffusion power in SIR model could be a good reason to be selected for ranking. Can other dimensionality reduction methods such as PCA give more precise results?
**Authors reply**: **This is an excellent suggestion to be used in future works. In this study, the implementation of the SIR model with many iterations to calculate the propagation power of nodes can indicate the reality of propagation in networks~~,~~ this model has been used in many articles.**

6.What do the Authorss mean by "making an attribute scale-free" in equation (2)?

**Authors reply:** Using an attribute scale-free made these features comparable, considering, different measurement scale of various attributes, so that the attributes can be measured without dimensions.

7. The Authors failed to explain why the ELECTRE method is chosen. The reader needs some more motivation.

**Authors reply**: I appreciate your consideration, in the Abstract and the proposed method section essential explanations, were added.
