# Supplementary material for: A multi-attribute method for ranking influential nodes in complex networks
Source: PLoS One. 2022 Nov 28;17(11):e0278129. doi: 10.1371/journal.pone.0278129 (PMC9704601; doi:10.1371/journal.pone.0278129)
Supplement: Attachment — Submitted filename: Response to Reviewers.docx [file pone.0278129.s002.docx]

**Manuscript Ref. No.: PONE-D-22-15682**

Dear Editor,

**Reply to reviewers on** PONE-D-22-15682

We would like first to thank the editor and the reviewers for helpful, constructive comments and observations that significantly contributed to improving the manuscript. Below we have concisely addressed each of the comments and suggestions and those changes implemented in the manuscript.

Also, we would like to note that the changes made in the text have been highlighted in blue in the new version of the manuscript to allow the reviewers to identify the newly added text easily.

Below you can find detailed explanations regarding each particular comment and the applied revision in the revised version of the manuscript.

Yours Sincerely,

Amir Sheikhahmadi, PhD

Assistant Professor, Islamic Azad University,

Sanandaj branch, Sanandaj, Iran

Email: sheikhahmadi@eng.ui.ac.ir

Reviewers' comments:

Reviewer #1:

1. The results of your comparative study should be discussed in-depth and with more insightful comments on the behavior of your algorithm on various case studies. Discussing results should not mean reading out the tables and figures once again.

**Authors reply:** Thanks for your valuable comment. In the revised version, All results were analyzed and added to the text.

2. Avoid lumping references as in [x, y] and all other. Instead summarize the main contribution of each referenced paper in a separate sentence. For scientific and research papers, it is not necessary to give several references that say exactly the same. Anyway, that would be strange, since then what is innovative scientific contribution of referenced papers? For each thesis state only one reference.

**Authors Reply:** proper changes were done in **the whole part of the manuscript.**

­

3. Avoid using first person.

**Authors reply:** Thanks for your consideration. It is done.

4. Avoid using abbreviations and acronyms in title, abstract, headings and highlights.

**Authors reply:** the proper modification is done **.**

5. Please avoid having heading after heading with nothing in between, either merge your headings or provide a small paragraph in between.

**Authors reply:** It is done.

6. There is The first time you use an acronym in the text, please write the full name and the acronym in parenthesis. Do not use acronyms in the title, abstract, chapter headings and highlights.

**Authors reply:** Thanks, It is done.

7. The results should be further elaborated to show how they could be used for the real applications.

**Authors reply:** Thank you, all the items were reviewed and corrected

Reviewer #2:

Almost all of the corrections suggested in the first round have been ironed out however, the manuscript needs extensive English editing because of there are typos and grammatical errors.

**Authors reply:** Thanks for your valuable comments which help us to improve manuscript. All sections have been revised carefully.

Reviewer #3:

I have no more comments.

**Authors reply:** Thanks for your valuable comments which help us to improve manuscript.
